# Lymphatic vasculature in the central nervous system

**DOI:** 10.3389/fcell.2023.1150775

**Published:** 2023-04-07

**Authors:** Sara González-Hernández, Yoh-suke Mukouyama

**Affiliations:** Laboratory of Stem Cell and Neuro-Vascular Biology, National Heart, Lung, and Blood Institute, National Institutes of Health, Bethesda, MD, United States

**Keywords:** blood-brain barrier, central nervous system, Prox1, meningeal lymphatics, lymphatic vessels, hybrid vessels

## Abstract

The central nervous system (CNS) is considered as an immune privilege organ, based on experiments in the mid 20th century showing that the brain fails to mount an efficient immune response against an allogeneic graft. This suggests that in addition to the presence of the blood-brain barrier (BBB), the apparent absence of classical lymphatic vasculature in the CNS parenchyma limits the capacity for an immune response. Although this view is partially overturned by the recent discovery of the lymphatic-like hybrid vessels in the Schlemm’s canal in the eye and the lymphatic vasculature in the outmost layer of the meninges, the existence of lymphatic vessels in the CNS parenchyma has not been reported. Two potential mechanisms by which lymphatic vasculature may arise in the organs are: 1) sprouting and invasion of lymphatic vessels from the surrounding tissues into the parenchyma and 2) differentiation of blood endothelial cells into lymphatic endothelial cells in the parenchyma. Considering these mechanisms, we here discuss what causes the dearth of lymphatic vessels specifically in the CNS parenchyma.

## 1 Introduction

In mammals the circulatory system is composed of two independent and complementary vascular networks that cooperate to maintain the proper functions in peripheral tissues and the organism fluid balance: while the blood vascular system is responsible for the transport of essential nutrients, hormones, and oxygen, as well as removal of waste across the body *via* the blood circulation, the lymphatic vascular system is a unidirectional route required to drain the interstitial fluid (ISF) extravasated from the blood capillaries and return it to the blood circulation, contributing thereby to the maintenance of tissue homeostasis. Additional well-known functions of the lymphatic system are the dietary lipid uptake in the gut and the mediation of immune cell trafficking and antigen presentation in the lymph nodes in charge of immune surveillance (Oliver and Alitalo, 2005; Tammela and Alitalo, 2010; Oliver et al., 2020; Petrova and Koh, 2020). The study of the lymphatic system is a rapidly evolving field, and the knowledge about its tissue-specific features and functions has greatly advanced in recent years.

Due to its relevant role to maintain tissue homeostasis and immune surveillance, lymphatic vasculature is typically found in most of the vascularized tissues, with the notable exception of the bone marrow, cartilage, and the central nervous system (CNS): we should note that, although it was believed that the bone and bone marrow lack lymphatic vessels, very recent work has revealed the presence of lymphatic vessels in bone, supporting bone and hematopoietic cell regeneration (Biswas et al., 2023). The lack of classical lymphatic network in the brain and spinal cord parenchyma limiting the ability to induce an immune response to CNS-derived antigens was the main reason why the CNS has been considered an immune-privileged organ (Shirai, 1921; Murphy and Sturm, 1923; Medawar, 1948). This concept was revised in the last decade with the discovery of the lymphatic-like hybrid vessels in the Schlemm’s canal in the eye (Aspelund et al., 2014; Kizhatil et al., 2014; Park et al., 2014; Truong et al., 2014; Kim et al., 2017) and the meningeal lymphatics in the dura mater, the outermost layer of the three meninges located under the skull (Aspelund et al., 2015; Louveau et al., 2015).

An additional feature that makes CNS tissue a unique immune privileged site is the presence of a physical blood-brain barrier (BBB), a heavily restringing barrier that regulates the CNS homeostasis, protecting the brain parenchyma from the entry of pathogens, toxins, circulating immune cells, and other factors to maintain proper neuronal activity (reviewed in Zhao et al., 2015; Obermeier, Daneman, and Ransohoff, 2013; Daneman and Prat, 2015). It is well-characterized that the BBB is a multicellular structure where the brain microvasculature is embedded in a unique environment known as “neurovascular unit,” where endothelial cells (ECs) are in close association with numerous pericytes, astrocytes, microglia, neurons, vascular smooth muscle cells and extracellular matrix (ECM). Altogether they form an impermeable barrier that maintains brain homeostasis by regulating the trafficking of fluid and solutes in both directions and therefore minimizing the potentially toxic compounds. CNS ECs are highly specialized compared with ECs in other tissues’ vasculature. Their special attributes include the control of paracellular and transcellular passage pathways through the expression of continuous tight junction (TJ) proteins, and restricted transcytosis and fenestration to avoid non-specific transcellular transport (Hawkins and Davis, 2005; Abbott et al., 2010; Obermeier, Daneman, and Ransohoff, 2013; Daneman and Prat, 2015; Iadecola, 2017). These properties allow the strict regulation of the movement of molecules, ions and cells between the blood and the CNS. In addition, brain ECs express low levels of leukocyte adhesion molecules (LAMs), consequently limiting the number of immune cells that enter in the CNS. Consistently, it has been widely described that decreased expression of TJ proteins or enhanced transcytosis rate is linked to the BBB breakdown, which is associated with pathological inflammation in the brain tissue and many neurodegenerative diseases, including stroke, multiple sclerosis, Alzheimer’s disease, and Parkinson’s disease. While these disorders have their own triggers, all of them converge in similar modifications of the CNS vasculature, suggesting that the BBB disruption is a common hallmark in these disorders, culminating in neuronal dysfunction, neuroinflammation and neurodegeneration (Hawkins and Davis, 2005; Obermeier, Daneman, and Ransohoff, 2013; Daneman and Prat, 2015; Zhao et al., 2015).

In this review, we discuss the unique anatomical aspects of the CNS vasculature, which include the presence of highly specialized ECs that comprise the BBB and the absence of a proper lymphatic system inside the brain and spinal cord parenchyma. We will debate whether the lack of lymphatic vessels in the CNS is developed by preventing 1) the invasion of lymphatic vessels from surrounding tissues (i.e., meningeal layers) and/or 2) the differentiation of CNS blood ECs into LECs. Considering that CNS blood ECs are specialized cells to form the BBB, we discuss the hypothesis that the lack of lymphatic differentiation potential of CNS blood ECs is tightly linked to their BBB formation. With a special interest in the absence of Prospero Homeobox protein 1 (Prox1) in CNS ECs (Antila et al., 2017; Izen et al., 2018), the key transcriptional regulator of lymphatic differentiation (Sabin, 1902; Wigle and Oliver, 1999; Srinivasan et al., 2007), here we provide new insights into the possible relationship between these two immune-privileged features.

## 2 An overview of the lymphatic vascular development

The lymphatic vasculature is a thin-walled and blind-ended system that transports the fluids and other components extravasated from the blood circulation (called lymph) through the lymph nodes back to the bloodstream (Wigle and Oliver, 1999; Tammela and Alitalo, 2010). Hierarchically, highly permeable initial lymphatic vessels characterized by the presence of interconnected and discontinuous button-like junctions between the lymphatic endothelial cells (LECs), and the absence of continuous basement membrane and perivascular mural cells facilitate the uptake of the ISF through passive drainage (Baluk et al., 2007). This ISF released from the blood vasculature is then transported through the pre-collecting and then collecting lymphatics, in which LECs are tightly connected with continuous zipper-like junctions (Baluk et al., 2007) where the presence of specialized perivascular smooth muscle cells induce contractile activity that promotes the unidirectional lymph flow (Muthuchamy and Zawieja, 2008; Norrmen et al., 2009; Alitalo, 2011; Bazigou and Makinen, 2013; Sabine et al., 2016).

The formation of the lymphatic network in mice begins during embryonic development, after a primitive but functional circulatory system is established. The first LEC progenitors are detected during embryonic days (E) 9.5–10.0, when a subset of venous ECs located in the jugular region of the cardinal vein starts to express Prox1 (Sabin, 1902; Wigle and Oliver, 1999; Srinivasan et al., 2007). Prox1 expression is initiated by Sox18 and Nr2f2 (also known as COUP-TF II) in embryonic venous ECs (Francois et al., 2008; Srinivasan et al., 2010). Prox1 transcription factor is widely considered as the master regulator of lymphatic vascular development, because its expression in blood ECs (BECs) induces the commitment toward a LECs fate by inducing LEC-specific transcriptional program as well as the repression of BEC-specific genes (reviewed in Oliver and Alitalo, 2005; Oliver and Srinivasan, 2010). Prox1+ LEC progenitors bud off from the cardinal vein, sprout and migrate to form the initial lymphatic plexus in response to vascular endothelial growth factor C (VEGF-C) (Karkkainen et al., 2004), which binds to its receptor VEGF receptor 3 (VEGFR3) expressed by the LECs (Dumont et al., 1998; Makinen et al., 2001; Karkkainen et al., 2004). The identity of LECs is maintained by a feedback loop between Prox1 and VEGFR3: Prox1 activates the expression of VEGFR3, and VEGF-C/VEGFR3 signaling regulates Prox1 (Srinivasan et al., 2014). The process of expansion of the initial lymphatic vessels by sprouting from pre-existing VEGFR3+ lymphatic capillaries in response to the morphogen VEGF-C is known as lymphangiogenesis (Vaahtomeri et al., 2017). When the primary lymphatic plexus has been established during embryonic development, it continues maturing postnatally to give rise to a hierarchical network of blind-ended capillaries, pre-collecting and collecting lymphatic vessels, characterized by the transformation from zipper-like to button-like junctions and the recruitment of smooth muscle cells that cover the collecting vessels. The development of lymphatic valves secures the unidirectional lymph flow whereas the lymphovenous valves restrict the entrance of the blood into the lymphatic vasculature. LECs can be distinguished from BECs based on their unique molecular signature. In addition to Prox1 and VEGFR3*,* LECs are distinguished by the expression of lymphatic vessels endothelial hyaluronan receptor 1 (LYVE-1) and glycoprotein podoplanin (PDPN) (Oliver and Srinivasan, 2010).

For decades, the origin of the lymphatic vasculature has been extensively debated. Consistent with the centrifugal theory described by Sabin in 1902 (Sabin, 1902), the major contribution from the venous vasculature that gives rise to the lymphatic network through lymphangiogenesis (sprouting of pre-existing vessels) (Vaahtomeri et al., 2017) was corroborated both in mice and zebrafish with a series of lineage tracing experiments and high-resolution imaging, indicating that the process is highly conserved across vertebrates (Sabin, 1902; Huntington and McClure, 1910; Yaniv et al., 2006; Srinivasan et al., 2007). Moreover, the most recent lineage tracing studies using the paraxial mesoderm specific *Pax3-Cre* and *Myf5-Cre* drivers demonstrated that the majority of LECs originate from the paraxial mesoderm-derived endothelium of the cardinal vein (Stone and Stainier, 2019). However, additional findings in recent years demonstrated that, while there is no doubt that the main source of lymphatics derives from the cardinal and intersomitic veins, further contribution from non-venous-derived progenitors, as described by McClure in the centripetal theory (Huntington and McClure, 1910) was found in specific tissues, in a process called lymphvasculogenesis (formation *de novo* of LECs). Examples of these non-venous origins are described as the contribution of local dermal blood capillary plexus to dermal lymphatics vasculature (Martinez-Corral et al., 2015; Pichol-Thievend et al., 2018), the contribution of second heart field cells and yolk sac hematopoietic derivatives to cardiac lymphatic vasculature (Klotz et al., 2015; Maruyama et al., 2019; Lioux et al., 2020) and the contribution of c-kit*+* hemogenic endothelium to mesenteric lymphatics (Stanczuk et al., 2015). In a similar fashion, studies in zebrafish using lineage-tracing and live imaging techniques reinforced the idea that LECs emerge from mixed venous and non-venous origins (Yaniv et al., 2006; Nicenboim et al., 2015; Eng et al., 2019). However, the roles of lymphatics from different sources in organ development and diseases are currently unclear.

## 3 The CNS is an immune privileged organ… devoid of lymphatic vasculature?

**FIGURE 1:**
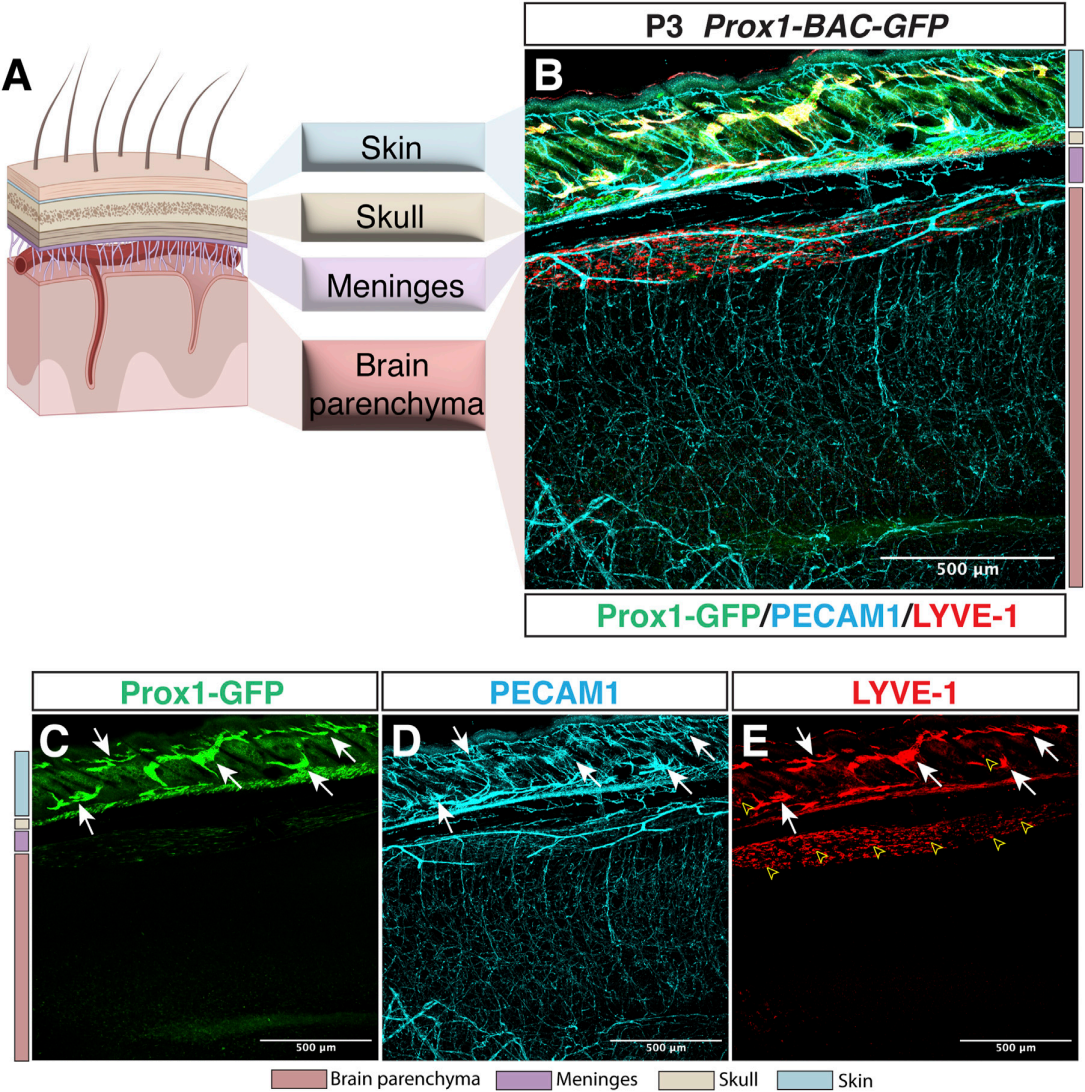
Lymphatic avascularity in the brain parenchyma. **(A)** Schematic representation of the brain, meningeal layers, skull bone, and skin on the left panel (Partially created with BioRender.com). **(B–E)** Whole-mount immunostaining of *Prox1-BAC-GFP* brain at postnatal day 3 (P3) is shown. Prox1-GFP labels lymphatic vessels in the skin [**(B,C)** green, arrows] as well as a subset of neural progenitors (not shown) in the brain parenchyma. All the vasculature is labeled with PECAM-1 [**(B,D)** cyan]. LYVE-1 labels lymphatic vessels [**(B,E)** red, arrows] and macrophages (open arrowheads) in the skin as well as the pia surface of the brain. Note that no lymphatic vessels are found in the brain parenchyma. Scale bar 500 μm.

Whilst the lymphatic system is present in most of the vascularized tissues in the body, the CNS comprises one of the few exceptions lacking lymphatic vessels within the brain and spinal cord parenchyma. Indeed, whole-mount imaging of *Prox1-BAC-GFP* mouse tissues (Choi et al., 2011) allows the direct identification of Prox1-expressing lymphatic vasculature *in vivo.* We here show a representative sagittal section image of *Prox1-BAC-GFP* mouse brain (Choi et al., 2011) at postnatal day 3 (P3) to illustrate the presence of lymphatic vessels (PECAM1+ Prox1-GFP+ LYVE-1+) in the skin but not inside the brain parenchyma (Figure 1). This feature in combination with the presence of a restrictive BBB provides an extremely unique environment for the immune cells, where immune surveillance is not achieved under physiological conditions.

During the early 20th century many experiments were performed transplanting grafts from a variety of tissues into anatomically unnatural sites in the body from the same or different species. Whereas in most of the cases the rejection from the host to the alien graft was observed, in some cases they could grow and survive, giving rise to the identification of “immunologically privileged” organs, including the anterior chamber of the eye, the cornea or the brain. The first evidence provided by Ebeling and Roffo is that allografts of a particular carcinoma were more successful when transplanted in the brain rather in the subcutaneous space using mice and rats, respectively (reviewed in Barker and Billingham, 1977). Xenografted rat sarcoma in the mouse brain could grow, whereas it was destroyed when placed subcutaneously or intramuscularly (Shirai, 1921). According to the observation that animals deprived of lymphoid tissue failed to destroy foreign tissue grafts, the brain could be an uncongenial environment for these immune cells, which in peripheral tissues were able to invade and destroy the tumors (Murphy and Sturm, 1923). Lastly, critical experiments carried out by Medawar described that, once primed peripherally, immune cells could induce an accelerated rejection against the foreign tissue, despite the brain’s status an immune-privileged site unable to induce an efficient adaptive immune response due to the lack of lymphatic drainage in this tissue (Medawar, 1948). Whereas in non-immunized animals the brain was unable to induce an immune response against the skin graft, in those animals where the skin was first grafted peripherally and then transplanted into the brain, a severe rejection was observed. These results showed that once peripherally activated, immune cells could migrate and enter the brain parenchyma, but the brain itself could not elicit such an immune response (Medawar, 1948).

**FIGURE 2:**
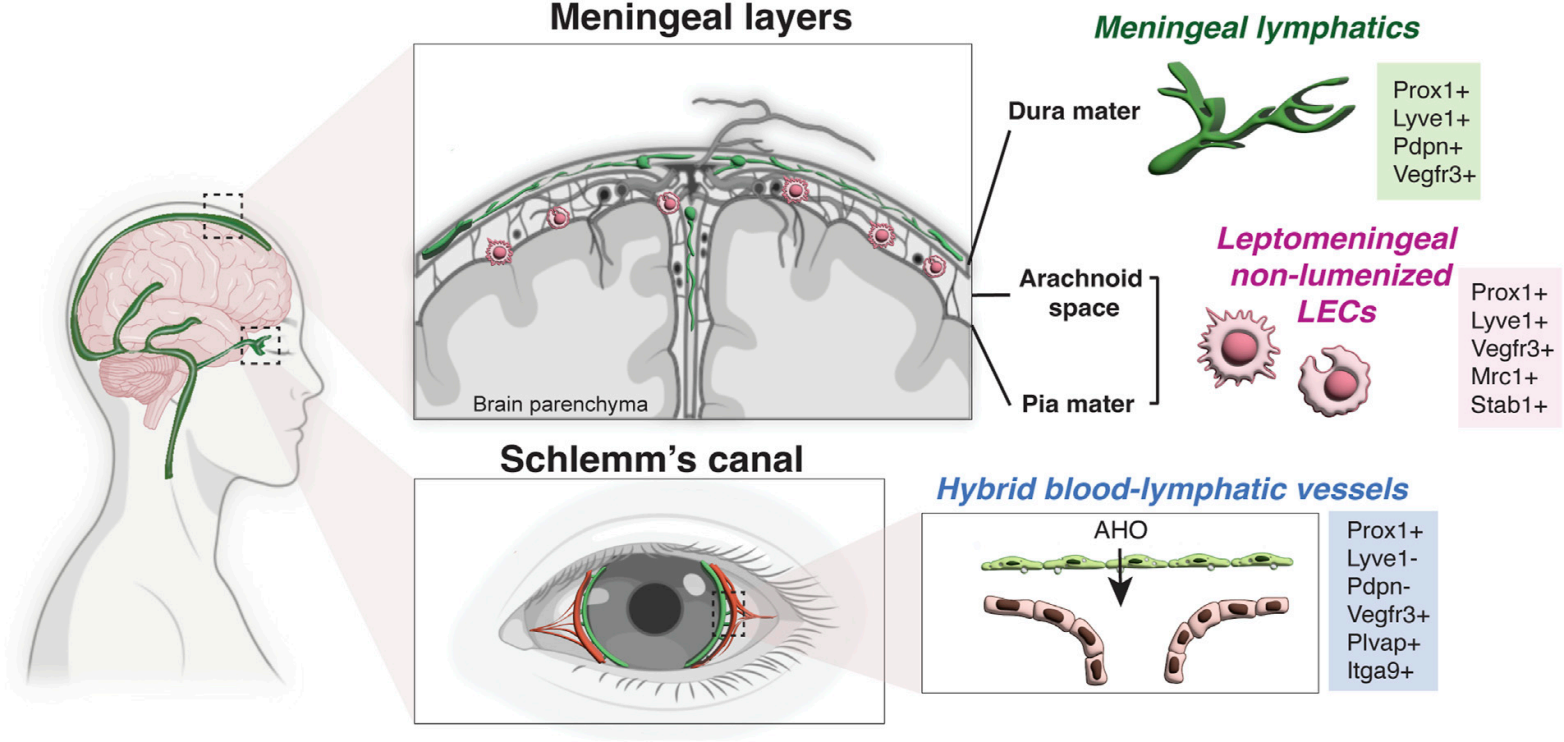
Lymphatic endothelial cells in the CNS. Schematic representation of a human brain showing the lymphatic endothelial cells in the meningeal layers and Schlemm’s canal. While there is no lymphatic vasculature in the brain parenchyma, meningeal lymphatic vessels are found in the dura mater under the skull and non-lumenized mural LECs are found in the leptomeninges (the arachnoid space and the pia mater). Meningeal lymphatics express the classical hallmark of lymphatic vessels (Prox1+ LYVE-1+ PDPN+ VEGFR3+), while non-lumenized mural LECs have macrophage-like shape and express the mannose receptor Mrc1 and Stab1. Note that Schlemm’s canal in the eye has hybrid blood-lymphatic vessels, expressing the lymphatic marker Prox1 but not LYVE-1 and PDPN. The hybrid vessels also express high level of VEGFR3, PLVAP and Itgα9. AHO, aqueous humor outflow (Partially created with BioRender.com).

Based on these transplant experiments, the eye and brain have been long considered immunologically unique and devoid of lymphatic vasculature; however this notion has been revised in the last decade due to the discovery of blood-lymphatic hybrid vessels in the Schlemm’s canal in the eye (Kizhatil et al., 2014; Park et al., 2014; Truong et al., 2014; Aspelund et al., 2015; Kim et al., 2017), the presence of classical lymphatic vessels in the meningeal dura mater of the brain (Aspelund et al., 2015; Louveau et al., 2015), and non-lumenized LECs in the surface of the brain in zebrafish and leptomeninges in mammals (Bower et al., 2017; van Lessen et al., 2017; Venero Galanternik et al., 2017; Shibata-Germanos et al., 2020). Although it is still maintained by the lack of classical lymphatic network inside the brain and spinal cord parenchyma, the concept of “immune privileged site” in the CNS has been recently redefined with these new insights (Figure 2).

### 3.1 Lymphatic-like vessels in the Schlemm’s canal in the eye

The Schlemm’s canal (SC) is a specialized ring-shaped vasculature at the periphery of the cornea which drains the aqueous humor outflow (AHO) from the intraocular chamber and delivers it back to the venous circulation to maintain fluid homeostasis in the eye. Malfunction in this drainage where the AHO is obstructed is frequently correlated with the development of glaucoma in humans. ECs lining the inner wall of the SC share morphologic and functional similarities with both BECs and LECs (Table 1). In addition to its vascular-derived origin, SC is a secondary structure that develops postnatally. Its ECs form a continuous monolayer lying on a discontinuous basement membrane, lack pericyte coverage, and establish a blind-ended tube that transports AHO and antigen-presenting cells in basal-to-apical flow to the systemic circulation. Altogether this special vasculature was considered more analogous to LECs rather than BECs, although initial studies did not examine the expression of LEC markers (reviewed in Ramos et al., 2007). The discovery of Prox1 expression in the SC ECs by different groups almost simultaneously was the milestone to consider this structure as hybrid vessels sharing blood and lymphatic properties but with lymphatic-like functions (Aspelund et al., 2014; Kizhatil et al., 2014; Park et al., 2014; Truong et al., 2014).

**TABLE 1:** Comparison between CNS endothelial cell types.

EC type	Key markers	Permeability	Supporting mural cells	References
BECs in non-CNS tissues	PECAM-1, EMCN, CD34, VEGFR1/2, PLVAP, ltga5	Capillaries, semipermeable	Pericytes in capillaries. Pericytes/SMCs in arteries/veins	Petrova et al. (2002), Johnson et al. (2008), Kim et al. (2010), Kim et al. (2013)
BBB ECs	PECAM-1, EMCN, Cldn5, Ocln, ZO-1, Mfsd2a, Sox 17, β-catenin	No permeable	NVU composed by BECs, pericytes and astrocytes	Liebner et al. (2008), Daneman et al. (2009), Corada et al. (2019), Zhou et al. (2014), Tran et al. (2016), Hussain et al. (2022), Ben-Zvi et al. (2014), Cui et al. (2021)
Meningeal LECs	PECAM-1, Prox1, LYVE-1, Pdpn, VEGFR3, ltga9, CCL21, Nrp2	Permeable. Basal mLVs mostly button-type junctions. Dorsal mLVs mostly zipper-type junctions	Pericytes/SMCs in collecting LVs. No pericytes/SMCs in capillaries LVs	Louveau et al. (2015), Aspelund et al., (2015), Antila et al. (2017), Ahn et al. (2019), lzen et al. (2018)
Hybrid ECs	PECAM-1, EMCN, PLVAP, ltga9, VEGFR3, Prox1, CCL21, CD34, FoxC2	Fenestrated/permeable. Disconinuous base membrane	No mural cells	Truong et al. (2014), Aspelund et al. (2014), Park et al. (2014), Kizhatil et al. (2014)
Mural LECs	Prox1, LYVE-1, VEGFR3, Mrc1, Stab1	Do not form tubes, macrophage-like morphology. Large cytoplasmatic vesicles	No mural cells	Bower et al. (2017), van Lessen et al. (2017), Venere Galanternik et al. (2017), Castranova et al. (2021), Shibata-Germanos et al. (2020)

SMCs, smooth muscle cells; NVU, neurovascular unit; BECs, blood endothelial cells; LEC, lymphatic endothelial cells; BBB, blood-brain barrier ; LVs, lymphatic vessels ; mLV, meningeal lymphatic vessels.

Despite the SC ECs expressing the lymphatic master regulator Prox1, they can be distinguished from classical lymphatic vasculature by the lack of other LEC markers such as LYVE-1 or PDPN (Aspelund et al., 2014; Truong et al., 2014) (Table 1), suggesting a partial lymphatic reprogramming. In a similar line of evidence, Sox18, the abovementioned upstream regulator of Prox1, was not detected in the SC ECs (Park et al., 2014). Moreover, SC ECs bud from the choroidal veins at postnatal day P1, and after the primordial SC is formed they upregulate the lymphatic master regulator Prox1 to acquire the hybrid lymphatic-blood phenotype (Park et al., 2014): SC expresses a mixture of BEC and LEC markers including the pan-endothelial platelet and endothelial cell adhesion molecule 1 (PECAM-1), the venous marker endomucin (EMCN), the chemokine (C-C motif) ligand 21 (CCL21), and VEGFR3, in addition to Prox1. They also express the lymphatic valve markers integrin alpha 9 (Itgα9) and Forkhead Box C2 protein (Foxc2), even though they do not have luminal valves (Aspelund et al., 2014; Park et al., 2014). Interestingly, abundant plasmalemma vesicle–associated protein (PLVAP), a marker for endothelial transcytosis and permeability, was also detected in the ECs of the inner SC wall (Figure 2; Table 1).

What controls the expression of Prox1 that is important for the SC integrity and functionality? Park et al. revealed that the shear stress-responsive transcription factor Klf4 might be an upstream regulator of Prox1 in SC ECs by directly binding to the first intron of the Prox1 gene. Likewise, a mouse ocular puncture model was used to confirm *in vivo* the requirement of flow to maintain the proper expression of Prox1. Indeed, reduction in AHO simultaneously downregulated Prox1 and Klf4 expression compared with control eyes (Park et al., 2014), proposing Prox1 as an accurate biosensor for SC functionality, which might be used for the early detection and prevention of glaucoma in humans.

Angiopoietin/Tie2 (Angpt/Tie2) signaling is required for the correct postnatal development and adult maintenance of the SC hybrid vessels to sustain the normal AHO (Thomson et al., 2014; Kim et al., 2017). Postnatal deletion of *Angpt1/Angpt2* or *Tie2* results in primary congenital glaucoma in mice due to increased intraocular pressure (Thomson et al., 2014). Meanwhile, Tie2-activating antibody (ABTAA) rescues the phenotype of glaucoma in double *Angpt1/Angpt2*-deficient mice (Kim et al., 2017), raising the possibility that this signaling pathway could be an interesting therapeutic target for the treatment of glaucoma.

### 3.2 The discovery of meningeal lymphatic vessels surrounding the brain

The surface of the brain is covered by three layers of meninges that comprise a superficial barrier between the cerebrospinal fluid (CSF) and the CNS: the dura mater is the outermost layer in contact with the skull bone, containing arteries, veins and fenestrated capillaries that do not form BBB; the arachnoid mater establishes a barrier between the dura and the subarachnoid space (SAS), which contains the CSF and resident immune cells; the pia mater is a monolayer of cells that covers the CNS separating it from the perivascular compartment (Engelhardt, Vajkoczy, and Weller, 2017). Given that no lymphatic vasculature exists in the CNS parenchyma (Figures 1, 2), the perivascular spaces of cerebral blood vessels have been proposed to serve as a pathway for the exchange of CSF and interstitial fluid to clear waste products (Iliff et al., 2012; Nedergaard, 2013). The recent identification of meningeal lymphatic vessels established another pathway for CSF outflow into deep cervical lymph nodes.

An extensive network of Prox1+ lymphatic vasculature was found in the dura mater (Figure 2), expressing hallmarks of classical LECs (PECAM-1, LYVE-1, PDPN, VEGFR3, CCL21) (Aspelund et al., 2015; Louveau et al., 2015; Antila et al., 2017; Izen et al., 2018; Ahn et al., 2019) (Figure 2; Table 1). Although the first evidence of the existence of lymphatics in the human dura mater was already described by Paolo Mascagni in 1787 (reviewed in Sandrone et al., 2019), due to their difficult location it is not until recently that the use of state-of-the-art confocal imaging and genetic models allowed a precise characterization and functional description of this lymphatic network surrounding the brain parenchyma.

In mice, multiple groups including ours, carried out an exhaustive examination of the meningeal lymphatic formation during postnatal and adult stages using *Prox1-BAC-GFP* mice (Antila et al., 2017; Izen et al., 2018). Whereas most lymphatic vessels in the peripheral tissues develop during embryonic stages, meningeal lymphatics are formed during the first postnatal month, appearing first at the base of the skull (Antila et al., 2017; Izen et al., 2018), as it was also reported in the Schlemm’s canal (Aspelund et al., 2014) and lacteal vessels in the gut (Kim, Sung, and Koh, 2007). Like classical lymphatic vessel development, meningeal lymphatics require Prox1 and VEGF-C/VEGFR3 signaling for their proper formation and maintenance during adult stages (Absinta et al., 2017). Indeed, blockade of this signaling impairs meningeal lymphatic development, while boosting VEGF-C induces meningeal lymphangiogenesis, suggesting promising regenerative potential (Aspelund et al., 2015; Antila et al., 2017; Hsu et al., 2019; Patel et al., 2019; Bolte et al., 2020; Song et al., 2020; Yanev et al., 2020). Nevertheless, a recent work showed that mice lacking *Plcγ2* have impaired structural remodeling and maturation of meningeal lymphatics, accompanied with reduced lymph flow, suggesting that meningeal lymphatic formation might not be only dependent on the VEGF-C/VEGFR3 signaling axis (Balint et al., 2019a). Intriguingly, after the re-discovery of the lymphatic vasculature in the dura mater in mice (Aspelund et al., 2015; Louveau et al., 2015), meningeal vessels were carefully examined in humans (Absinta et al., 2017; Eide et al., 2018; Jani and Sekula, 2018; Shibata-Germanos et al., 2020; Jacob et al., 2022), non-human primates (Absinta et al., 2017), and zebrafish (Castranova et al., 2021). Meningeal lymphatic vasculature that wraps the spinal cord also develops postnatally, through VEGF-C/VEGFR3 signaling (Antila et al., 2017; Louveau et al., 2018; Jacob et al., 2019).

The presence of immune cells in the meningeal vessels under the steady state suggests their involvement in immune cell trafficking and immunosurveillance of the CNS (Louveau et al., 2015). Recent studies elegantly demonstrated the skull and vertebrae bones as new bone marrow niches for hematopoiesis supplying blood-borne myeloid cells (including monocytes, neutrophils, and macrophages) and B cells to the dura mater (Brioschi et al., 2021; Cugurra et al., 2021; Schafflick et al., 2021). These cells transit from the bone to the meninges through specialized vascular connections, challenging the previous accepted idea that meningeal adaptative immunity originates from the systemic circulation.

High-resolution imaging with the injection of fluorescent tracers into the brain parenchyma or the CSF in the cisterna magna clearly demonstrated that lymphatic vessels in the dura mater play an essential role in the clearance of macromolecules and immune cells from the CSF within the SAS into the deep cervical lymph nodes (dcLNs) (Aspelund et al., 2015; Louveau et al., 2015; Absinta et al., 2017; Da Mesquita et al., 2018; Louveau et al., 2018; Ahn et al., 2019; Balint et al., 2019b; Bolte et al., 2020; Chen et al., 2020). Interestingly, there are anatomical differences in meningeal vasculature between the dorsal and basal part of the skull. While dorsal meningeal lymphatics mainly present zipper-like junctional pattern of LECs, in basal vessels the LECs are loosely joined by intermittent button-like junctions, which allow them a better uptake of the CSF macromolecules and subsequent drainage outside the brain (Ahn et al., 2019). Altogether, these recent findings about key roles of meningeal lymphatics in the CNS clearance and immunosurveillance under physiological conditions generated substantial excitement in medical research due to their potential contributions in a variety of pathological models, including autoimmune diseases, cerebrovascular injury, brain tumors or age-related neurological diseases, which implied promising avenues for the therapeutic treatment of cerebrovascular injuries and age-related neurodegenerative diseases.

Extraordinary work by Jacob et al. showed a similar anatomy in the meningeal lymphatic vasculature between mice and humans (Jacob et al., 2022). 3D characterization with unprecedented resolution was performed combining the imaging of intact whole head in mice and improved non-invasive magnetic resonance imaging (MRI) in humans, filling gaps in the knowledge about the vascular connections between the meninges and the collecting lymph nodes. More importantly, the innovative use of MRI combining elliptic venography, T1 SPACE and DANTE sequences provided enough resolution to compare the meningeal lymphatic network in human patients and identify morphological anomalies. As an example, the authors examined 11 patients with neurovascular diseases and identified that only those with Gorham-Stout disease had significantly altered meningeal lymphatics. Thus, this work provides new insights in the detection of meningeal alterations between individuals with neuropathological conditions (Jacob et al., 2022).

### 3.3 Non-lumenized LECs in the surface of zebrafish brain and mammalian leptomeninges: Potential roles in neurological diseases and regeneration

Recently, multiple groups described the presence of a distinctive LEC population in the surface of the brain (Figure 2). These LECs appear to be unusual isolated perivascular cells with a macrophage-like morphology, and they do not form lumenized tubes under physiological conditions, although they express Prox1 and exhibit features of LECs due to their molecular markers (LYVE-1, VEGFR3) and their venous derived origin, which is dependent on VEGF-C/VEGFR3 signaling (Bower et al., 2017; van Lessen et al., 2017; Venero Galanternik et al., 2017; Castranova et al., 2021).

Initially identified in the zebrafish model, brain perivascular LECs are known as mural LECs (muLECs) (Bower et al., 2017), brain LECs (bLECs) (van Lessen et al., 2017) or fluorescent granular perithelial cells (FGPs) (Venero Galanternik et al., 2017). Interestingly, an analogous population was also found in the mouse and human leptomeninges, the brain-associated meningeal layers that includes the arachnoid mater and pia mater, which is why they were termed leptomeningeal LECs (LLECs) (Shibata-Germanos et al., 2020). In mammals, FGPs or “Mato Cells” were previously characterized as perivascular macrophages with a bone marrow-derived origin (Mato and Ookawara, 1981; Audoy-Remus et al., 2008; Faraco et al., 2016) due to the expression of the Mannose Receptor 1 (Mrc1) and their scavenger potential, which was thought to protect the brain from toxic waste products by phagocytosis and pinocytosis (reviewed in Suarez and Schulte-Merker, 2021). Nevertheless, studies with extensive live imaging and sophisticated lineage tracing discard the pericytes-, neural crest- or hematopoietic-derived origins, demonstrating instead that this unique population derives from pre-existing venous vessels in the CNS (Bower et al., 2017; van Lessen et al., 2017; Shibata-Germanos et al., 2020), which sprout to cover the surface of the brain before acquiring their mesenchymal/perivascular morphology (Bower et al., 2017). In a similar fashion, genetic deletion of the myelopoietic lineage in *PU.1* mutants did not affect the formation of these muLECs/bLECs/FGPs/LLECs (van Lessen et al., 2017; Shibata-Germanos et al., 2020), but the loss of *Ccbe1,* a VEGF-C-activating protease, gave rise to abnormal sprouting and asymmetrical distribution of these cells on the surface of the brain (van Lessen et al., 2017), clearly demonstrating their lymphatic origin and dependence on VEGF-C/VEGFR3 signaling.

A key hallmark of perivascular LECs is the presence of large cytoplasmic vesicles and the expression of scavenger receptors Mrc1 and Stab1 (Table 1), suggesting their endocytic role to uptake and clear waste products that enter into the interstitial space for degradation. Previous studies with tracer injections in the parenchyma or CSF in mouse and zebrafish showed the ability of perivascular LECs to actively take up polysaccharides and glycoproteins with at least 150 KDa size and internalize them through endocytosis with higher efficiency than macrophages or microglia, whereas lower efficiency was proved in the uptake of molecules higher than 500 KDa (Venero Galanternik et al., 2017; Shibata-Germanos et al., 2020; Huisman et al., 2022). Such endocytic capacity quickly raised the interest in examining whether they could play a relevant role in the surveillance and clearance of Aβ peptides in the brain, the toxic driver of Alzheimer Disease’s progression. Real-time *in vivo* analysis in zebrafish allows the investigation of the clearance capacity of Amyloid-β isoform 42 (Aβ_1–42_). Remarkably, Jeong et al. (2021) demonstrated that the dynamic uptake of Aβ_42_ depends on its aggregation status, where Aβ_1-42_ monomers are selectively internalized and cleared by perivascular LECs but not Aβ_1–42_ oligomers. The discovery of these isolated LECs in the surface of the brain involved in the homeostasis and uptake of Aβ peptides provides new therapeutic avenues in AD, where the enhancement of their function in combination with the reduction of Aβ aggregates might contribute to new approaches to prevent their accumulation. Similarly, Amyloid β_1–40_ clearance by LLECs was shown in mice, illustrating their conserved functions in vertebrates (Shibata-Germanos et al., 2020).

Under physiological conditions, muLECs/bLECs/FGPs/LLECs stay as loose single cells without forming lumenized lymphatic vessels; however, elegant studies by Chen et al. (2019) described sticking roles for perivascular LECs in resolving tissue damage caused by vascular dysfunction in the zebrafish brain. Using a model of brain cerebrovascular injury in zebrafish, the authors demonstrated that some perivascular LECs could form lymphatic tubes that penetrated the brain parenchyma to drain the ISF and resolve the edema. Although such transient invasion of lymphatic vessels has not been reported in mammals, it demonstrates a potential role of perivascular LECs in brain regeneration in the zebrafish model.

## 4 Potential mechanisms of CNS lymphatic avascularity

The continuous emerging of clearing tissue protocols in combination with the use of 3D imaging from intact tissues with cell-specific markers is revolutionizing the identification of lymphatic vasculature in tissues that were long considered devoid of them, including the brain and spinal cord in the CNS, the eye, and the bone. The most recent example was the sighting of lymphatic vessels inside the bone in mice and humans (Biswas et al., 2023). However, in the CNS, despite the finding of lymphatic vessels and non-lumenized LECs in the meningeal layers surrounding the surface of the brain (dura mater and leptomeninges, respectively), there is no evidence of the presence of classical lymphatic vasculature inside the brain parenchyma in mammals under physiological conditions.

Potential mechanisms that could promote the appearance of lymphatic vessels in the different organs are 1) LEC sprouting and invasion of pre-existing lymphatic vessels (lymphangiogenesis) in response to lymphangiogenic signaling such as VEGF-C/VEGFR3, or 2) differentiation of BECs or non-ECs into LECs (lymphvasculogenesis), which requires the expression of the lymphatic transcriptional regulator Prox1. We discuss how the CNS develops lymphatic avascularity by preventing LEC invasion and differentiation.

### 4.1 LEC invasion into the CNS parenchyma in the zebrafish vasculature model

Multiple reports have suggested the boosting of VEGF-C signaling as a promising therapeutic approach to enhance meningeal lymphangiogenesis to halt pathologies like edemas, or toxic peptides accumulation (Aspelund et al., 2015; Antila et al., 2017; Hsu et al., 2019; Patel et al., 2019; Bolte et al., 2020; Song et al., 2020; Yanev et al., 2020). Nevertheless, lymphatic vessels or non-lumenized LECs have not been observed inside the brain and spinal cord parenchyma in mammals under steady-state or disordered conditions. Still the molecular cues that might prevent the CNS environment from the invasion of meningeal lymphatics remain unelucidated. Similarly, an extensive population of LYVE-1+ macrophages are found in the borders of the brain, but never inside the parenchyma (Figure 1E). LYVE-1 is a well-established receptor for hyaluronan (HA), a key component of the extracellular matrix in many tissues (Banerji et al., 1999). Considering the recent studies that LYVE-1+ cells are associated with HA-enriched regions in the mammary gland (Wang et al., 2020a), HA accumulation in the boundaries of the brain and spinal cord may function as a physical barrier to prevent the invasion of LYVE-1-expressing cells. Further studies would be required to elucidate such a mechanism underlying lack of LYVE-1-expressing cells in the CNS parenchyma.

In addition to the apparent incapability of meningeal lymphatics to penetrate the brain parenchyma in mammals, a transient lymphatic invasion of the abovementioned non-lumenized LECs in response to cerebrovascular injury was described in zebrafish model. By using NTR-Mtz system and photochemical thrombosis to induce brain vascular injury, Chen et al. (2019) proved the capability of LECs located in the borders of zebrafish brain to activate the ingrowth of LECs upon cerebrovascular injury, dependent on VEGF-C/VEGFR3 signaling, and penetrate the injured brain parenchyma to drain the ISF and resolve brain edema. In this work, the authors classified the ingrowth lymphatic vessels (iLVs) in two different subpopulations depending on their functions: “stand-alone iLVs” and “track iLVs.” The stand-alone iLVs become lumenized lymphatics to drain the ISF and resolve the edema in the injured brain parenchyma whereas the other iLVs transdifferentiate into blood vessels (iLVs-to-BVs transdifferentiation), giving rise to early-formed blood vessels in the ischemic area. The track iLVs serve as a migratory scaffold (or “growing tracks”) to guide and support the growth of late-generated blood vessels necessary to promote the functional recovery in the damaged tissue without inducing uncontrolled vessel growth (Chen et al., 2019; Chen et al. 2021; Chen et al., 2022). Mechanistically, iLV-to-BV transdifferentiation exclusively occurs in stand-alone iLVs through activation of Notch signaling (Chen et al., 2019). These new early formed blood vessels are covered by pericytes and become functional with blood flow within 2 days after injury. By contrast, track iLVs are unable to transdifferentiate since Notch signaling is suppressed. Surrounding blood vessels express *ephrinB2a*, activating the expression of *EphB4* in the track iLVs, which in turn promotes Notch inhibition (Chen et al., 2021). Moreover, the authors described that the formation of new vessels guided by the “growing tracks” is regulated by CXCL12/CXCR4 signaling axis (Chen et al., 2022). Whilst the chemokine receptor *Cxcr4a* is transcriptionally activated in track iLVs after cerebrovascular damage, its ligand *Cxcl12b* is expressed by the surrounding blood vessels, guiding the directionality of iLV ingrowth. By using *Cxcr4a* and *Cxcl12b* mutants, the authors highlighted the critical role of CXCL12/CXCR4 signaling in controlling the appropriate directionality and vessel patterning during cerebrovascular regeneration, avoiding increased branching and uncontrolled formation of blood vessels (Chen et al., 2022). This is a transient invasion of lymphatic vessels inside the brain parenchyma, in that stand-alone iLVs gradually disappear after the completion of cerebrovascular regeneration by apoptosis, maintaining the lymphatic avascular status inside the brain parenchyma once the brain tissue is fully recovered (Chen et al., 2021).

### 4.2 No lymphatic differentiation in the CNS vasculature

The transcription factor Prox1 is involved in many developmental processes including neurogenesis and tissue development such as heart, eye lens, liver, and pancreas. In the vascular system, it serves as the “master regulator” that controls the switch from BEC into LEC fate. During the last two decades, a series of pioneering studies showed that Prox1 is required for the acquisition and maintenance of the lymphatic phenotype (Wigle and Oliver, 1999; Hong et al., 2002; Petrova et al., 2002; Johnson et al., 2008; Kim et al., 2010; Kim et al., 2013). The expression of Prox1 in the LEC progenitors that bud off from the cardinal vein is necessary not only to activate the expression of LEC-specific genes, but also to repress BEC-specific transcriptional programs (Johnson et al., 2008). Numerous *Prox1* loss-of-function and gain-of-function studies clearly demonstrated the malleability of ECs to acquire BEC or LEC fate. *In vivo* and *in vitro* assays clearly showed that Prox1 is necessary and sufficient to induce the lymphatic phenotype and repress the BEC program (Wigle and Oliver, 1999; Hong et al., 2002; Petrova et al., 2002; Francois et al., 2008; Johnson et al., 2008). Likewise, in the absence of this transcription factor the suppression of the BEC program is not achieved and ECs lose their lymphatic identity (Johnson et al., 2008; Kim et al., 2010; Kim et al., 2013).

Considering the lack of Prox1 expression in the CNS vasculature, in this section we discuss the hypothesis that Prox1 expression might be strictly controlled in the CNS ECs to prevent LEC differentiation and preserve the BBB properties. Here we provide a general overview about regulatory mechanisms of Prox1 expression in the lymphatic system, and we debate potential mechanisms that might regulate Prox1 expression in the CNS vasculature, with a particular focus on Wnt/β-catenin signaling required for the BBB development and maintenance.

#### 4.2.1 Prox1 regulation in LEC development

**FIGURE 3:**
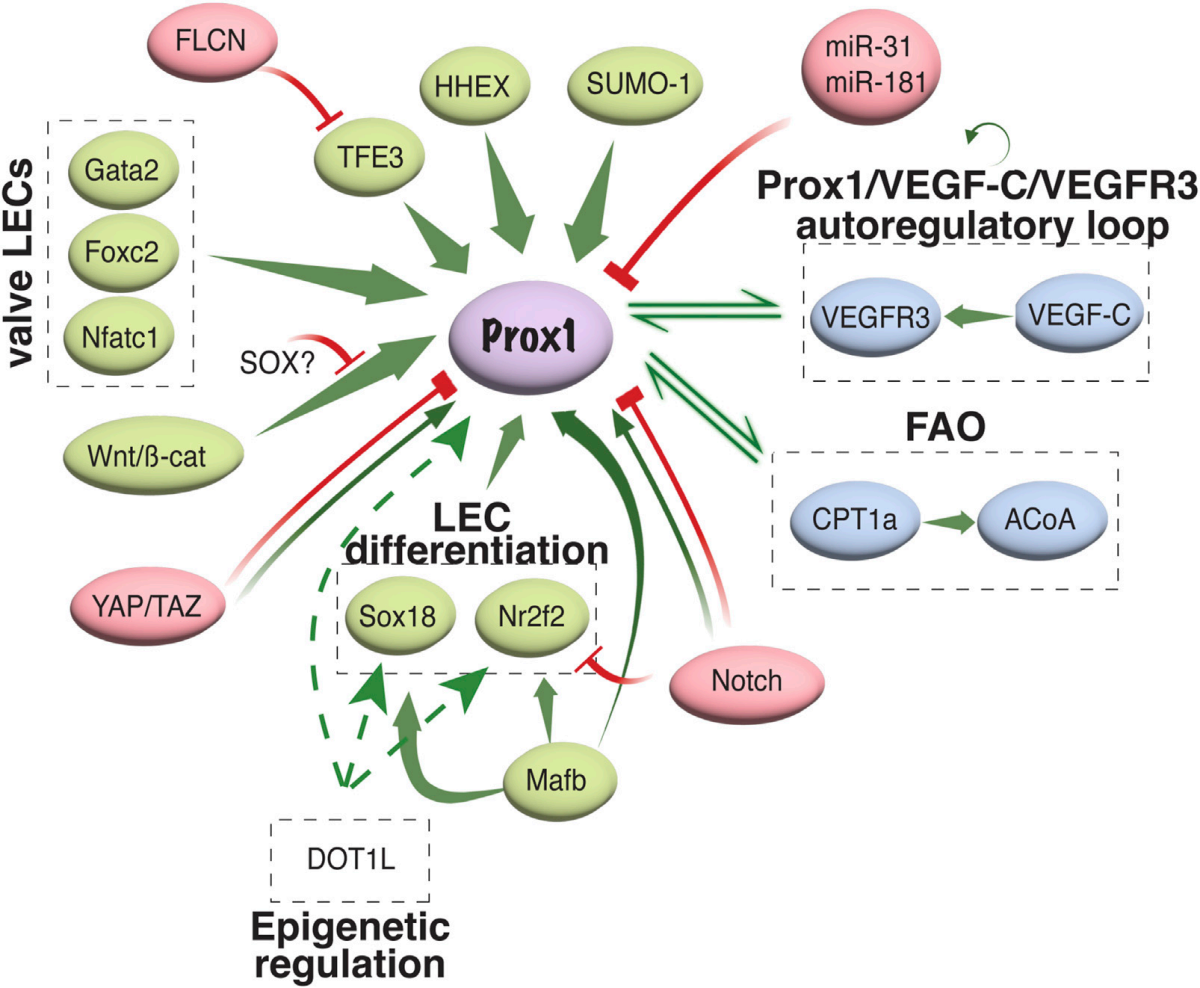
Transcriptional and epigenetic regulation of Prox1 in the vascular system. Schematic representation of different activators/repressors of Prox1 expression in the vascular system is shown. FAO, fatty acid oxidation; ACoA, acetyl coenzyme A.

**FIGURE 4:**
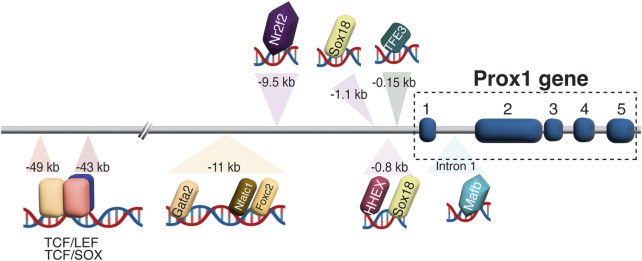
Prox1 regulation in LEC development. Schematic representation of biding motifs for multiple transcriptions factors and regulators in the upstream of the Prox1 gene is shown. In the −43/−49 kb region, TCF:LEF and TCF:SOX overlapping binding sites are found, suggesting a possible competition between LEF and SOX factors to bind to the same motif.

Diverse transcriptional and epigenetic regulators have been proposed to control Prox1 expression in the vascular system (summarized in Figure 3). The initiation of Prox1 expression in the LEC progenitors requires the expression of Sox18 and Nr2f2 transcription factors (Francois et al., 2008; Srinivasan et al., 2010). While Sox18 is only needed for the initial specification of LECs (Francois et al., 2008), Nr2f2 is critical for the venous specification, which is required for the subsequent lymphatic specification and migration of these progenitors away from the cardinal vein (Srinivasan et al., 2007; Srinivasan et al., 2010), but not for the maintenance of the lymphatic fate once the lymphatic system has been established. A binding motif for Nr2f2 was found in the −9.5 kb upstream open reading frame of the *Prox1* gene, whereas two different binding sites have been described for Sox18 (−1.1 and −0.8 kb, respectively, Figure 4). Epigenetically, the histone H3K79 methyltransferase DOT1L controls Sox18 transcriptional regulation, which subsequently regulates Prox1 expression, as well as other lymphatic essential genes such as Foxc2 and VEGFR3, by inducing chromatin opening that promotes their transcriptional activation (Yoo et al., 2020). Mafb is another upstream regulator of the lymphatic differentiation factors Prox1, Sox18 and Nr2f2 during embryonic and postnatal development (Dieterich et al., 2015). By directly binding to the first intron in the *Prox1* gene, the combination Mafb and Prox1 generates a positive feedback loop in which the increased expression of Prox1 also maintains the expression of Mafb. In a similar line of work, another well-known positive feedback loop in the lymphatic vasculature is the Prox1/VEGF-C/VEGFR3 axis. The Prox1-VEGFR3 autoregulatory loop is critical for controlling the number of specified LEC progenitors budding from the cardinal vein in response to VEGF-C and the subsequent maintenance of the lymphatic identity (Srinivasan et al., 2014). Furthermore, the hematopoietically expressed homeobox (HHEX) transcription factor was recently found as an upstream regulator of Prox1 as well as VEGF-C and FLT4 during lymphatic development. Indeed, HHEX binds to the −0.8 kb upstream of the *Prox1* transcriptional start site (Gauvrit et al., 2018) (Figures 3, 4).

An interesting regulatory region including the binding sites of Gata2, Foxc2, Nfatc1, and Prox1 itself was identified in the −11 kb upstream of the *Prox1* transcriptional start site. Oscillatory shear stress from the lymph flow upregulates the levels of these factors, which therefore binds to the −11 kb enhancer region to increase its expression in the valvular LECs (Kazenwadel et al., 2015). Genome editing of the GATA-binding site in this enhancer element abolished its activity and impaired lymphatic vascular development, demonstrating the crucial role of GATA2 in activating the Prox1 −11 kb regulatory element (Kazenwadel et al., 2023). Moreover, the authors revealed that mutants with a reduced expression of Prox1 showed an enhanced hemogenic capacity, submitting the first evidence of lymphatic endothelium as a source of hematopoietic cells, and suggesting the role of Prox1 in repressing this property (Kazenwadel et al., 2023). Wnt/β-catenin signaling also interacts with Prox1 in a feedback loop to induce the expression of Gata2 and Foxc2 in the valvular ECs (Cha et al., 2016). Folliculin (FLCN) suppresses the lymphatic specification in venous ECs through the degradation of TFE3 transcription factor, which binds to the −0.15 kb upstream of the Prox1 transcription start site, and therefore prevents the expression of the lymphatic master regulator: lack of *folliculin* allows TFE3 to translocate into the nucleus and upregulate Prox1 expression by binding to its promoter in venous ECs (Tai-Nagara et al., 2020) (Figures 3, 4).

Suppression of Prox1 expression has been proposed to control the balance between LEC and BEC specification. *In vitro* studies suggest that *miR-31* inhibits lymphatic fate by downregulating Prox1 expression (Pedrioli et al., 2010). Similarly, *miR-181* downregulates Prox1 expression by binding to its 3-UTR region, which leads to its translational inhibition and transcript degradation (Kazenwadel, Michael, and Harvey, 2010). Notch signaling functions as a negative regulator of the Nr2f2/Prox1 transcriptional axis to block lymphatic specification (Kang et al., 2010; Zheng et al., 2011; Murtomaki et al., 2013; Choi et al., 2017). By contrast, opposite results were found in zebrafish and postnatal mice, where Dll4/Notch signaling is required for the correct lymphatic development (Geudens et al., 2010; Niessen et al., 2011). These contrary roles of Notch signaling in regulating LEC fate could be context-dependent to maintain the precise balance between BEC and LEC specification. In a similar line of research, YAP/TAZ signaling functions as a positive regulator or inhibitor of Prox1 expression in the different studies (Cho et al., 2019; Cha et al., 2020). Whereas Cho et al. (2019) found that YAP and TAZ act as important negative regulators of Prox1 during lymphatic remodeling and postnatal lymphatic valve morphogenesis, Cha et al. (2020) described thar YAP/TAZ signaling was required to maintain the valvular ECs and experiments *in vitro* demonstrated that YAP/TAZ positively regulates Prox1 in primary human LECs (HLECs). The discrepancies found between both studies might be due to the differences in the genetic models used, nevertheless both groups obtained the same results that the hyperactivation of YAP/TAZ signaling results in the downregulation of Prox1 *in vitro*. These results suggested that a proper level of YAP and TAZ is required to maintain the expression of Prox1 in LECs (Figure 3).

Post-translational regulations of Prox1 influence the transcriptional response (reviewed in Bui and Hong, 2020). Several lysine sites on Prox1 can be modified by small ubiquitin-related modifier (SUMO) (Shan et al., 2008). Sumoylation modulates Prox1 DNA binding to their target genes, enhancing the transcriptional activation of downstream lymphatic-related genes (Pan et al., 2009). In cancer cells, the Ser79 phosphorylation of Prox1 by AMP-activated protein kinase promotes Prox1 degradation through the recruitment of the CUL4-DDB1 E3 ubiquitin ligase complex (Wang et al., 2022). Despite the importance in post-translational regulations to modulate Prox1 transcriptional activity, how different modifications of Prox1 in response to distinct upstream signals regulate LEC development and maintenance remain elusive.

Lastly, novel findings spotlighted the involvement of metabolism in the regulation of Prox1 and lymphangiogenesis. A beautiful work by Wong et al. described a role of fatty acid β-oxidation (FAO) in LEC differentiation. Prox1-mediated transcriptional upregulation of carnitine palmitoyltransferase 1a (CPT1a) promotes mitochondrial-dependent FAO which increases the level of acetyl coenzyme A (ACoA). The ACoA is utilized by Prox1/p300 complex to promote the histone acetylation of key lymphangiogenic genes, including *Prox1* itself. Therefore, the upregulation of CPT1a by Prox1 is also a positive feedback loop to maintain Prox1 expression active in LECs (Wong et al., 2017). Moreover, recent exciting study by Ma et al. demonstrated that mitochondrial complex III is required to maintain the epigenetic regulation that promoted the Prox1-VEGFR3 autoregulatory loop (Ma et al., 2021).

Altogether, the expression levels of Prox1 in the vascular system encompass multiple transcriptional and epigenetic players that maintain the proper balance between blood endothelial and lymphatic endothelial specification.

#### 4.2.2 Specific regulation of Prox1 in the CNS vasculature

**FIGURE 5:**
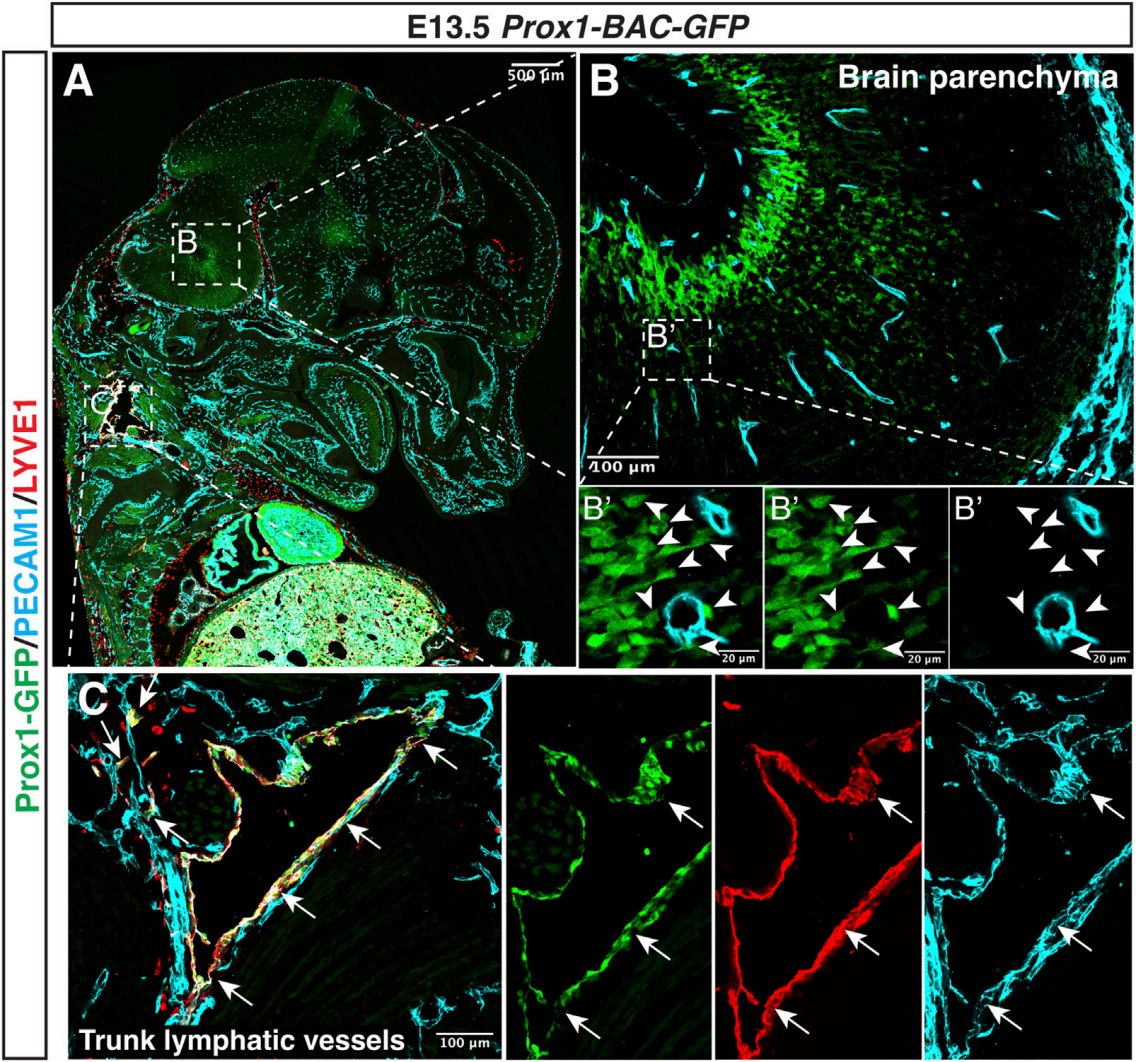
Prox1 is not expressed in the developing CNS vasculature. Section immunostaining of *Prox1-BAC-GFP* brain at E13.5 with anti-PECAM1 (cyan) and anti-LYVE-1 (red) is shown. The boxed regions in **(A)** are magnified in **(B,C)**, and the boxed region in **(B)** is magnified in **(B’)**. In the brain parenchyma **(B,B’)**, Prox1-GFP expression is found in a subset of neural progenitors [**(B)** green, white arrowheads], but this GFP expression does not colocalize with the PECAM1+ endothelial cells [**(B’)** green vs. cyan]. In the trunk **(C)**, Prox1-GFP + LYVE-1+ PECAM1+ lymphatic vessels are found [**(C)** arrows]. Scale bars 500, 100, and 20 μm.

Although some Prox1+ LECs are derived from venous capillaries in the developing Schlemm’s canal and meninges, no Prox1 expression is observed in the vasculature of the brain and spinal cord parenchyma (Antila et al., 2017; Izen et al., 2018) (Figure 5). Considering the observation that *in vivo* overexpression of Prox1 in the vascular bed using *Tie1* promoter decreased the expression of junctional proteins such as zonula occludens 1 (ZO-1), Occludin (Ocln) and JAM-1, as well as triggered increase permeability (Kim et al., 2010; Kim et al., 2013), the lack of lymphatic vessels and suppression of Prox1 in ECs might be compatible with the BBB function which regulates the brain homeostasis and protects the CNS. CNS ECs might avoid the expression of Prox1 to maintain the BBB properties and functions; however, little is known about whether ectopic Prox1 expression affects the BBB properties and functions and what regulates Prox1 expression in the CNS ECs.

The canonical Wnt/β-catenin signaling is essential for the acquisition and maintenance of the BBB properties (Liebner et al., 2008; Daneman et al., 2009). Activation of T-cell factor/lymphoid enhancer factor (TCF/LEF1) in brain ECs is required to control the expression of BBB-specific programs that induce the expression of TJ proteins and the repression of PLVAP to inhibit fenestration, permeability, and transcytosis. Given that Wnt/β-catenin signaling declines at late embryonic and postnatal stages (Corada et al., 2019), Wnt/β-catenin signaling appears to be maintained at the minimum active levels to maintain the integrity of the adult BBB: EC-specific deletion of *β-catenin* in adult mice compromises the BBB integrity by downregulating the expression of TJ proteins Claudin 5 (Cldn5), ZO-1 and Ocln (Zhou et al., 2014; Tran et al., 2016; Hussain et al., 2022). Moreover, these mutants increase transcytosis due to a reduction in the expression of major facilitator superfamily domain containing 2A (Mfsd2a), which represses the caveolae-dependent transcytosis (Ben-Zvi et al., 2014; Andreone et al., 2017; Wang et al., 2020b; Cui et al., 2021; Hussain et al., 2022).

Studies by Boye et al. (2022) described the transmembrane receptor Unc5b and its ligand Netrin-1 as upstream regulators of β-catenin in brain ECs, since their absence provokes cerebrovascular leak in postnatal and adult mice. Endothelial-specific deletion of *Unc5b* in mice generated BBB leakage for tracers up to 40 KDa and the brain microvasculature and displayed decreased Cldn5 and increased PLVAP expression, common features under the BBB breakdown.

Interestingly, there is wide evidence that Prox1 is a downstream target of Wnt/β-catenin/TCF signaling in many different cell types, including colon cancer cells, neural stem cells (NSCs), LECs, lymphatic valves, and hematopoietic stem cells (HSCs) (Petrova et al., 2008; Karalay et al., 2011; Nicenboim et al., 2015; Cha et al., 2016; Cha et al., 2018; Lin et al., 2022). TCF/LEF binding sites are identified in the −43 and −49 kb regions of the *Prox1* transcriptional start site, which are conserved in human, mouse, rat, and chicken (Petrova et al., 2008; Karalay et al., 2011) (Figure 4). ChIP assay demonstrated that TCF4 binds specifically to the −49 kb region to activate Prox1 expression in intestinal cancer cells (Petrova et al., 2008), whereas in murine hippocampal NSCs, Prox1 activation requires the binding of TCF/LEF to both the −43 and −49 kb regions (Karalay et al., 2011). The canonical Wnt/β-catenin signaling regulates LEC specification in zebrafish and human embryonic stem cells (hESCs) (Nicenboim et al., 2015) by promoting the angioblast-to-lymphatic transition, as well as inducing Prox1 expression in HSCs (Lin et al., 2022).

The Sry-related HMG box 17 (Sox17) transcription factor exerts a positive role on Wnt/β-catenin signaling in CNS ECs to maintain the BBB (Corada et al., 2019) and blood-retina barrier (BRB) properties (Yang et al., 2020). Sox17 is one of the major transcriptional targets of Wnt/β-catenin signaling during CNS vascular development, while Sox17 modulates Wnt/β-catenin signaling. Indeed, EC-specific deletion of Sox17, which causes BBB disruptions, can be rescued by the expression of a constitutive active form of β-catenin (Corada et al., 2019). Considering that Sox17 expression increases during development and after birth, Sox17 and β-catenin act in synergy to maintain the integrity of the brain microcirculation. Interestingly, TCF/LEF and SOX binding sites overlap in the −43 and −49 kb regions upstream *Prox1* locus (Figure 4), indicating that the presence of SOX family proteins may repress the effects of Wnt/β-catenin on *Prox1* transcriptional activation (Kuwabara et al., 2009; Karalay et al., 2011).

Altogether, although the regulation of the lymphatic master regulator Prox1 in the CNS vasculature is poorly understood, there is indirect evidence that supports that its expression should be downregulated in the brain and spinal cord ECs to keep the BBB properties, which include the high expression of TJ proteins, lack of fenestration and low rate of transcytosis and permeability across the barrier. Future studies to determine whether SOX family transcription factors expressed in the brain microvasculature could be inactivating the effect of Wnt/β-catenin signaling on Prox1 locus to control its expression as it has been already shown in adult neurogenesis (Kuwabara et al., 2009) would be interesting.

## 5 Concluding remarks and outstanding questions

Traditionally, the CNS has been considered an immune-privileged site due to two main features that differentiate this tissue from the others in the body: 1) the lack of efficient immune response, which is linked with the absence of lymphatic vasculature in the brain and spinal cord parenchyma, and 2) the presence of a restricted blood-brain barrier composed by unique microvasculature, characterized by the expression of high level of TJ proteins and restricted transcytosis and fenestration, properties required to maintain the barrier function. Considering the functional and structural differences between the lymphatic and the CNS vascular beds, the specialization of the CNS ECs to form the BBB is incompatible with the acquisition of the lymphatic phenotype in the brain and spinal cord parenchyma. But how does the CNS microenvironment prevent lymphatic vessel development?

We here discuss two potential mechanisms by which the CNS prevents the emergence of lymphatic vasculature inside the brain and spinal cord parenchyma. One potential mechanism is that, albeit the extensive network of meningeal lymphatics in the skull-associated meninges and non-lumenized LECs in the surface of the brain, the CNS may prevent the LEC sprouting and invasion from the pre-existing lymphatic vessels in adjacent tissues. The other is that the CNS may prevent the differentiation of BECs into LECs, through the suppression of Prox1 expression in CNS ECs.

It seems that the CNS microenvironment provides a permissive environment for the development of blood vasculature but not lymphatic vasculature. VEGF-A is expressed and released by neural progenitors to promote the ingrowth of capillaries in the developing brain and spinal cord parenchyma. Likewise, VEGF-C is also expressed in embryonic and early postnatal brain and induces proliferation of neural progenitors (Le Bras et al., 2006; Ward and Cunningham, 2015). However, CNS ECs express lower level of VEGFR3, possibly due to the lack of the positive feedback loop between Prox1 and VEGFR3. These observations suggest that the lack of lymphatic vessels in the brain and spinal cord parenchyma is unlikely to result from an insufficient microenvironment for lymphatic vessel development.

VEGF-C/VEGFR3 signaling activates lymphangiogenesis in development as well as pathological settings. In the bone, the tissue long considered devoid of lymphatic vasculature until the recent findings (Biswas et al., 2023), the exogenous expression of VEGF-C was enough to induce lymphangiogenesis and invasion of pre-existing lymphatic vessels in the neighbor tissues (Hominick et al., 2018; Monroy et al., 2020). Similarly, taking advantage of the plasticity shown by the lymphatic system, several groups promoted the expansion of meningeal lymphatics by the exogenous expression of VEGF-C, resulting in a better immune response against brain tumors or improvement of CSF drainage to reduce neuroinflammation (Antila et al., 2017; Song et al., 2020).

Brain mural LECs in the zebrafish vasculature model invade into the brain parenchyma and become lumenized lymphatics that can drain the ISF and resolve the edema (Chen et al., 2019). However, in the murine CNS vasculature model, LEC invasion from the meningeal lymphatic vessels or leptomeningeal non-lumenized LECs into the brain parenchyma has not been reported under the steady or pathological conditions. Further studies are necessary to elucidate the impact of this change during evolution.

The transcriptional and epigenetic regulators controlling Prox1 expression in the vascular system maintain the balance between BEC and LEC fate; nonetheless, little is known about specific Prox1 regulation in the CNS BECs. There are examples of non-CNS organs where Prox1 expression is prevented to ensure the segregation between blood and lymphatic vasculatures. Findings from Tai-Nagara et al. (2020) highlighted the role of FLCN gene as a gatekeeper to avoid Prox1 expression in those BECs that must keep the blood specification. The expression of FLCN in BECs prevents the translocation of the TFE3 to the nuclei, impairing the activation of Prox1 and therefore the induction of lymphatic specification.

What is the link between the lack of lymphatic vessels and BBB formation? The forced expression of Prox1 in BECs leads to a reduced expression of junctional proteins and an increase in the vascular permeability (Kim et al., 2010; Kim et al., 2013). In the CNS vasculature, these changes are incompatible with the maintenance of the BBB integrity, raising the possibility that Prox1 expression should be tightly suppressed in BBB ECs. Which signaling controls Prox1 suppression and BBB formation? Wnt/β-catenin signaling is crucial for the acquisition and maintenance of the BBB properties, including the induction of TJ and adherens junction proteins as well as the inhibition of fenestration, permeability, and transcytosis. Of note, identification of TCF/LEF binding sites upstream of the *Prox1* locus suggested this transcription factor as a downstream target of Wnt/β-catenin/TCF pathway in different cell types. In a similar line of research, Sox17 is a major downstream target and regulator of Wnt/β-catenin signaling, and TCF/LEF and SOX binding sites overlap in the −43 and −49 kb regions upstream *Prox1* locus. Whether inactivation of Wnt/β-catenin signaling and Sox17 affects Prox1 expression in CNS ECs remains unclear. Elegant studies in zebrafish by Das et al. (2022) demonstrated that anal fin vascularization requires transdifferentiation of previously formed lymphatic vessels into blood vessels. Notably, the loss of LEC fate in this model is related to the upregulation of several BEC markers, where Sox17 plays a relevant role in the suppression of Prox1 expression to promote the LEC-to-BEC transition. Considering that Sox17 expression increases in CNS ECs during BBB development and maturation, Sox17 might repress LEC fate and maintain CNS EC properties. Further studies will provide the fundamental mechanism of lymphatic avascularity in the brain and spinal cord parenchyma. Moreover, a possible dysregulation of Prox1 expression could lead to BBB alterations in pathological scenarios.
